# A genome-wide association study for late-onset Alzheimer's disease using DNA pooling

**DOI:** 10.1186/1755-8794-1-44

**Published:** 2008-09-29

**Authors:** Richard Abraham, Valentina Moskvina, Rebecca Sims, Paul Hollingworth, Angharad Morgan, Lyudmila Georgieva, Kimberley Dowzell, Sven Cichon, Axel M Hillmer, Michael C O'Donovan, Julie Williams, Michael J Owen, George Kirov

**Affiliations:** 1 Department of Psychological Medicine, Cardiff University School of Medicine, Heath Park, Cardiff, CF14 4XN, UK; 2 Biostatistics & Bioinformatics Unit, Cardiff University School of Medicine, Heath Park, Cardiff, CF14 4XN, UK; 3 Department of Genomics, Life & Brain Center, University of Bonn, Sigmund-Freud-Strasse 25, D-53127 Bonn, Germany

## Abstract

**Background:**

Late-onset Alzheimer's disease (LOAD) is an age related neurodegenerative disease with a high prevalence that places major demands on healthcare resources in societies with increasingly aged populations. The only extensively replicable genetic risk factor for LOAD is the apolipoprotein E gene. In order to identify additional genetic risk loci we have conducted a genome-wide association (GWA) study in a large LOAD case – control sample, reducing costs through the use of DNA pooling.

**Methods:**

DNA samples were collected from 1,082 individuals with LOAD and 1,239 control subjects. Age at onset ranged from 60 to 95 and Controls were matched for age (mean = 76.53 years, SD = 33), gender and ethnicity. Equimolar amounts of each DNA sample were added to either a case or control pool. The pools were genotyped using Illumina HumanHap300 and Illumina Sentrix HumanHap240S arrays testing 561,494 SNPs. 114 of our best hit SNPs from the pooling data were identified and then individually genotyped in the case – control sample used to construct the pools.

**Results:**

Highly significant association with LOAD was observed at the *APOE *locus confirming the validity of the pooled genotyping approach.

For 109 SNPs outside the *APOE *locus, we obtained uncorrected p-values ≤ 0.05 for 74 after individual genotyping. To further test these associations, we added control data from 1400 subjects from the 1958 Birth Cohort with the evidence for association increasing to 3.4 × 10^-6 ^for our strongest finding, rs727153.

rs727153 lies 13 kb from the start of transcription of lecithin retinol acyltransferase (phosphatidylcholine – retinol O-acyltransferase, *LRAT*). Five of seven tag SNPs chosen to cover *LRAT *showed significant association with LOAD with a SNP in intron 2 of *LRAT*, showing greatest evidence of association (rs201825, p-value = 6.1 × 10^-7^).

**Conclusion:**

We have validated the pooling method for GWA studies by both identifying the *APOE *locus and by observing a strong enrichment for significantly associated SNPs. We provide evidence for *LRAT *as a novel candidate gene for LOAD. *LRAT *plays a prominent role in the Vitamin A cascade, a system that has been previously implicated in LOAD.

## Background

Late-onset Alzheimer's disease (LOAD) is an age related neurodegenerative disease and the most common form of dementia in the over 65 age group. It affects 20% of people aged 75 – 84 years, rising to 50% in the over 85's, thus placing major demands on healthcare resources in societies with increasingly aged populations [1]. It has a high heritability with estimates ranging between 60 – 80% [2]. The only extensively replicable genetic risk factor for LOAD is the apolipoprotein E gene, in which the ε4 genotype is overrepresented in LOAD cases compared to controls. The ε2 genotype is underrepresented and believed to have a protective effect on disease development. However the presence of the *APOE*-ε4 genotype is neither necessary nor sufficient for the development of the disease, indeed 40 – 70% of European LOAD patients do not carry an ε4 variant, and additional genetic loci remain to be identified [3].

Recent advances in genotyping technology make it possible to conduct genome-wide association (GWA) studies, testing the whole genome with hundreds of thousands of single nucleotide polymorphisms (SNPs). For a complex disease such as LOAD, in which multiple genetic and environmental factors are thought to contribute to risk [2,4] GWA studies offer the potential to detect susceptibility genes with greater confidence than with linkage analysis. It has been estimated that over 80% of genetic variation of common SNPs of the human genome in European populations can be captured at an *r*^2 ^> 0.8 by using current SNP genotyping arrays [5].

In order to detect variants of small effect, particularly if the association is indirect, and to overcome the issue of multiple testing, large sample sizes are required [6,7]. Currently, GWA studies are expensive, generally restricting this type of work to groups or consortia with substantial funding for that purpose. Genome-wide association analysis of pools of case and control DNA offers an economic approach with the potential to identify disease loci [8-10]. In DNA pooling, equal amounts of DNA from each sample are combined to form pools from cases and controls, which are genotyped to get an estimate of the true allele frequency difference for each variant. This estimate is then used to test a limited number of SNPs for genetic association at a fraction of the cost of individual genotyping [9,11,12].

In this study, 561,494 SNPs were genotyped in DNA pools constructed from the Medical Research Council (MRC) Genetic Resource for LOAD case – control samples. In order to select SNPs for testing by individual genotyping, we applied three complimentary approaches to select the highest-ranked SNPs. We successfully genotyped individually 114 SNPs. We found association with LOAD of several SNPs close to the *APOE *locus (2.08 × 10^-9 ^– 8.24 × 10^-11^) thus confirming the validity of the pooled genotyping approach. In addition, we obtained evidence for several novel genetic associations to LOAD, our most significant findings being association of SNPs in the lecithin retinol acyltransferase (phosphatidylcholine – retinol O-acyltransferase, *LRAT*) gene.

## Results

Before we started work with Illumina arrays, we validated the pool construction with the SNaPshot method. The pools gave an accurate estimate of the real difference in allele frequencies for 3 SNPs previously individually genotyped in this sample. rs11084424, rs157580 and rs157581 showed allele frequency differences between cases and controls in pools of 4%, 9% and 17% respectively which compared well with the real differences of 3%, 10% and 19%. Published estimates of the accuracy of DNA pooling report similar high accuracy with errors of < 2% between predicted and true allele frequency differences using different methods [11].

**Figure 1:**
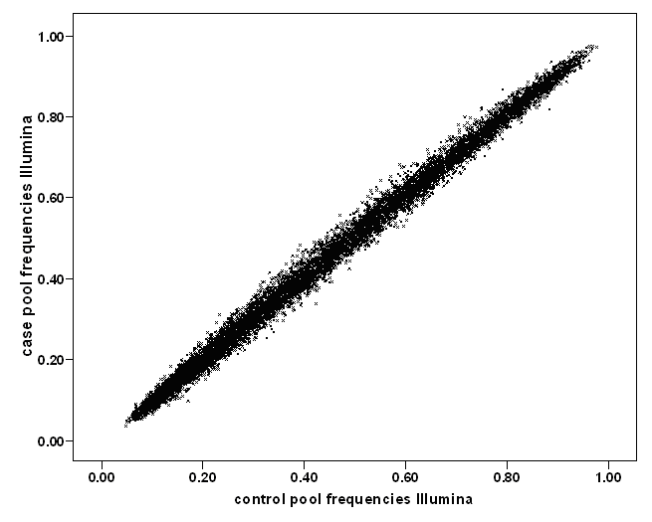
**Scatter plot of pooled genotype data**. Predicted allele frequencies of ~31,000 randomly selected SNPs in LOAD case and control DNA pools predicted by the Illumina HumanHap300 array. Averaging three case and four control arrays, we obtain a correlation r = 0.998.

**Figure 2:**
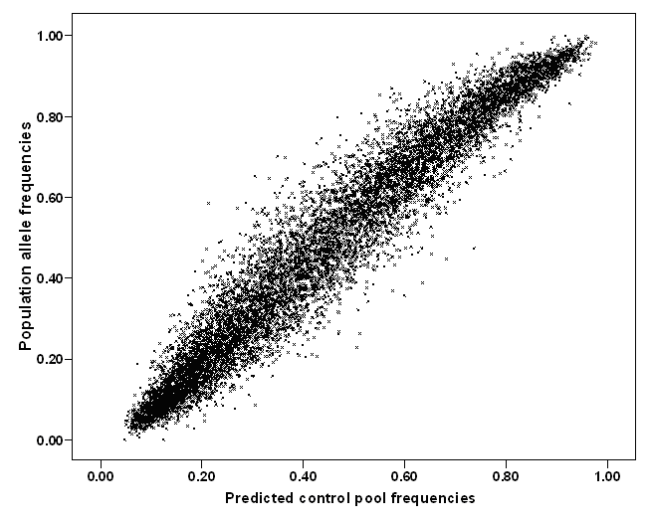
**Scatter plot of pooled vs individual (population) genotype data**. Predicted allele frequencies were averaged across technical replicates for the control pool and compared to actual population frequencies determined from the 1958 Birth Cohort, r = 0.969.

Genome-wide pooled genotyping was carried out on the Illumina HumanHap300 and Illumina Sentrix HumanHap240S arrays assaying 561,494 SNPs. Frequencies for each SNP were averaged over four replicate case and three replicate control arrays for the Illumina HumanHap300 and eight each for the Sentrix HumanHap240S arrays. The predicted averaged patient and control allele frequencies showed as expected a very high Pearson correlation with each other of r = 0.998, indicating a low technical variability of the method. Figure 1 shows predicted allele frequencies in case and control pools determined using the Illumina HumanHap300 platform. Data from the Illumina Sentrix HumanHap240S arrays showed similarly high correlations (r = 0.997). Predicted allele frequencies were compared with actual population allele frequencies (1958 Birth Cohort controls genotyped with the same HumanHap300) and gave a very good correlation of 0.969 (Figure 2). This indicates that even uncorrected data from pooling on this platform predict fairly well the true absolute allele frequencies of SNPs.

**Figure 3:**
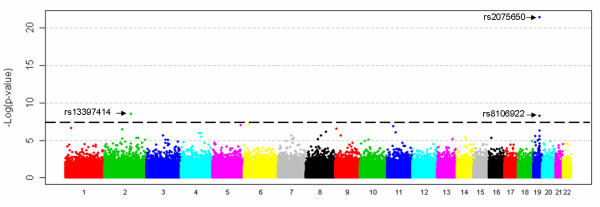
**Plot of combined Z-test p-values against chromosomal location for pooled data**. Out of 561,494 SNPs that were genotyped in our case – control pools, only 3 would have remained significant after Bonferroni correction for multiple testing, two of these SNPs are near the *APOE *gene on Chromosome 19. For a genome-wide association study on 500,000 markers (assuming that markers are independent) the significance level is 10^-7^. The line of genome-wide significance is plotted therefore at this level, although the combined Z-statistic provides only an approximation of the p-values produced from individual genotyping.

**Figure 4:**
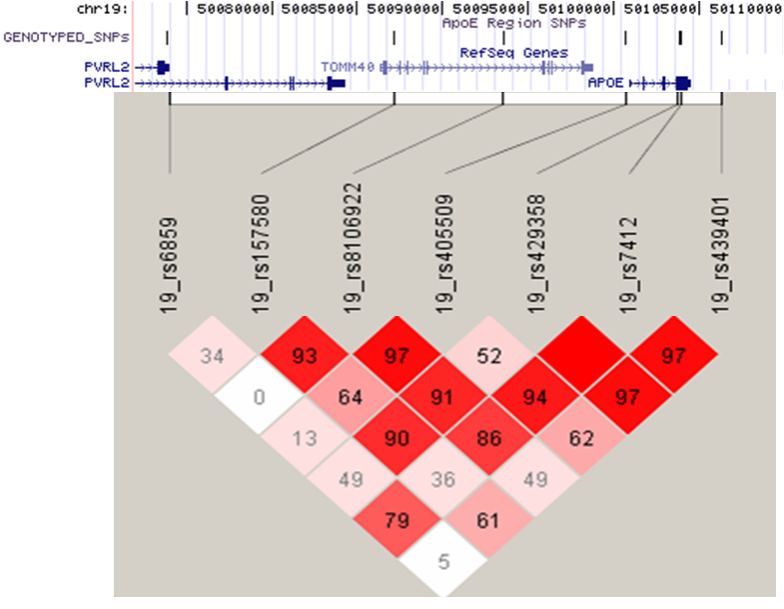
**LD plot for SNPs in the region of *APOE***. SNPs in the region of *APOE*, significant from Illumina pooled genotyping were individually genotyped and show high LD (D' given) with SNPs that define *APOE*-ε2/ε3/ε4 status (rs429358 and rs7412).

Figure 3 shows the combined Z-test p-value results for the whole genome. On the X-axis we have plotted the position in the genome by chromosome, and on the Y-axis the negative logarithm of the p-value. The strongest evidence for association with LOAD was observed with SNPs on chromosome 19 surrounding the *APOE *gene. In all, 7 SNPs within 71 kb were predicted by the pools to have allele frequency differences between 6% – 14% and "combined" p-values ranging from 9.0 × 10^-5 ^to 3.6 × 10^-22^. No other region of the genome showed such a large number of significant markers over a relatively small region. Five of the seven SNPs were individually genotyped and were confirmed to be highly significant (p-value range 2.08 × 10^-9 ^– 8.24 × 10^-11^, Table 1). All are in high linkage disequilibrium (LD) with the SNPs that define the *APOE *genotypes which are not themselves typed on the Illumina platform (Figure 4). The distributions of *APOE *alleles in this population are as follows:

**Table 1:** Individually genotyped SNPs that show significant association with LOAD.

SNP ID	Chrom	Position	Gene Symbol	SNP Type	Minor Allele	Pooled fcas	Pooled fcon	Ind fcas	Ind fcon	Ind f 1958	Comb Z-test pools	p Cases vs MRC Cons	p Cases vs All Cons
Rs6859 α	19	50073874	PVRL2	T/C	A	0.423	0.324	0.519	0.419	0.417	6.00E-07	1.73E-10	6.09E-14
Rs157580 α	19	50087106	TOMM40	A/G	G	0.653	0.583	0.693	0.595	0.616	6.43E-05	8.24E-11	3.87E-11
Rs8106922α	19	50093506	TOMM40	A/G	G	0.622	0.507	0.698	0.599	0.596	5.89E-09	1.21E-10	3.96E-14
Rs405509 α	19	50100676	APOE	A/C	G	0.547	0.468	0.558	0.463	0.521	7.33E-06	2.08E-09	4.77E-06
Rs439401 α	19	50106291	APOE	T/C	T	0.303	0.372	0.276	0.361	0.357	8.97E-05	7.97E-09	9.15E-11
Rs727153 ‡	4	156012026	intergenic	T/C	C	0.425	0.515	0.475	0.540	0.535	0.0009	0.00002	3.37E-06
Rs3754675	2	101008361	NPAS2	T/C	C	0.814	0.741	0.939	0.905	0.936	5.70E-06	0.00003	0.0144
Rs4699852	4	95699967	intergenic	T/C	A	0.392	0.468	0.431	0.493	0.451	0.0008	0.00004	0.0032
Rs2905990	5	11160513	CTNND2	A/G	T	0.298	0.369	0.300	0.356	0.327	3.79E-05	0.00009	0.0014
Rs9600764	13	76208794	intergenic	T/C	G	0.904	0.860	0.974	0.953	0.964	9.98E-05	0.00019	0.0027
Rs1032412	2	163464614	KCNH7	A/G	G	0.536	0.484	0.726	0.673	0.711	0.0024	0.00019	0.0097
Rs12510838	4	73326573	NPFFR2	A/G	G	0.627	0.558	0.828	0.783	0.810	0.0008	0.00024	0.0047
Rs7140253	14	106305044	intergenic	T/C	G	0.772	0.711	0.898	0.862	0.875	3.18E-05	0.00024	0.0007
Rs1455280	4	61600197	intergenic	T/C	G	0.870	0.820	0.900	0.864	0.882	0.0001	0.00027	0.0026
Rs13397414	2	154056922	intergenic	A/G	G	0.529	0.428	0.895	0.859	0.867	3.44E-09	0.00028	0.0003
Rs587259	19	39348246	LSM14A	T/C	T	0.515	0.450	0.397	0.344	0.364	0.0002	0.00028	0.0009
Rs13115107	4	183493069	intergenic	T/C	G	0.466	0.537	0.687	0.736	0.722	0.0001	0.00032	0.0005
Rs1373900	2	193561888	intergenic	T/C	T	0.259	0.322	0.329	0.380	0.346	5.38E-05	0.00037	0.0087
Rs1911014	4	127131703	intergenic	A/G	A	0.145	0.205	0.124	0.162	0.155	3.56E-06	0.00037	0.0003
Rs17228994	5	14095376	intergenic	A/C	C	0.668	0.736	0.849	0.885	0.870	3.60E-05	0.00060	0.0025
Rs407146	16	13223156	LOC729993	A/C	T	0.294	0.239	0.416	0.365	0.399	0.0007	0.00060	0.0137
Rs7937776	11	94336790	HSPC148	A/G	C	0.576	0.642	0.797	0.837	0.826	0.0006	0.00067	0.0007
Rs3819902	21	42973084	PDE9A	A/G	C	0.623	0.555	0.914	0.883	0.887	0.0009	0.00068	0.0004
Rs16916856	8	52901703	PCMTD1	T/C	C	0.745	0.677	0.887	0.852	0.868	7.80E-06	0.00069	0.0036
Rs393195	19	49184982	ZNF155	A/C	G	0.600	0.525	0.760	0.715	0.745	0.0002	0.00071	0.0147
Rs12146414	10	17108257	CUBN	A/G	G	0.829	0.759	0.933	0.905	0.920	1.37E-05	0.00086	0.0067
Rs7798395	7	70326814	WBSCR17	T/C	C	0.691	0.777	0.923	0.948	0.941	2.20E-06	0.00090	0.0011
Rs741477 ‡	2	64977962	intergenic	A/G	G	0.852	0.795	0.898	0.867	0.858	5.44E-05	0.00124	3.05E-05
Rs10161338‡	12	114328023	intergenic	T/C	C	0.759	0.682	0.902	0.872	0.875	3.52E-05	0.00176	0.0009
Rs10406335‡	19	49325793	ZNF225	A/G	G	0.637	0.571	0.905	0.878	0.876	0.0003	0.00390	0.0009
Rs9324088 ‡	14	106041164	intergenic	T/C	G	0.616	0.544	0.751	0.718	0.699	0.0003	0.01295	0.0002

Pooled fcas: Predicted Allele Frequency in MRC Case Pools. Pooled fcon – Predicted Allele Frequency in MRC Control Pools

Ind fcas: Actual Allele Frequency in MRC Cases.

Ind fcon: Actual Allele Frequency in MRC Controls.

Ind f1958: Allele Frequency in 1958 Birth Cohort

Comb Z-test pools: the p-value of combined Z-test for allelic association in pooled data.

p Cases vs MRC Cons: refers to p-value for allelic association in LOAD case subjects vs LOAD MRC control subjects matched for age, gender ethnicity.

p Cases vs All_Cons: refers to p-value for allelic association in LOAD case subjects vs MRC LOAD control subjects combined with genotype data obtained from the 1958 Birth Cohort

α : SNPs in the region of *APOE*

‡ : SNPs that show an approximately 10 fold increase in significance when additional controls from the 1958 Birth Cohort are combined with controls from the MRC sample (p Cases vs All Cons)

*APOE*-ε2, *APOE*-ε3, *APOE*-ε4 = 5.1, 57.9, 37.0 and 9.5, 77.3, 13.2% in cases and controls respectively, giving an allelic p-value of p = 1.9 × 10^-73 ^and an odds ratio (95%CI) = 3.85 (3.55 – 4.15) for the *APOE*-ε4 allele.

Table 1 shows results for our most significant SNPs, in addition to those at the *APOE *locus, following individual genotyping. The most significant SNP, rs727153, reached a p-value of 2.4 × 10^-5^.

To further test the associations, we added control data from a set of controls comprising approximately 1400 subjects from the 1958 Birth Cohort for the 79 SNPs that showed individual genotype p-value ≤ 0.05. The association for five SNPs becomes more significant with the inclusion of the additional controls. The majority of SNPs however became less significant indicating that for these SNPs we had probably identified false positives, although this could also happen to true-positive findings, as the initial discovery study tends to over-estimate the effect size. Our strongest finding, rs727153 remained the most significant individually genotyped SNP, with the evidence for association increasing from 2.4 × 10^-5 ^to 3.4 × 10^-6^.

**Figure 5:**
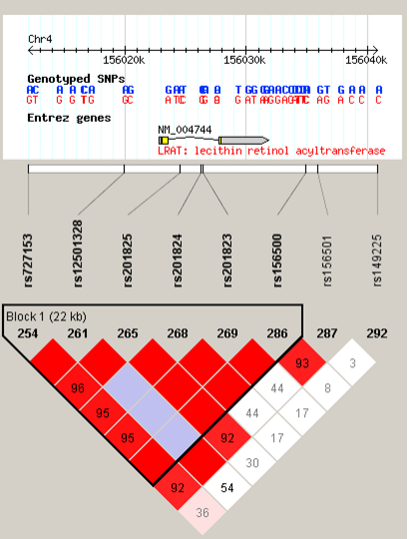
**LD plot for SNPs in the region of *LRAT***. Linkage Disequilibrium plot (D' values shown) for tagSNPs chosen to cover an LD block containing *LRAT*. The most significant SNP from GWA study, rs727153, is in high LD with SNPs in *LRAT*.

rs727153 is an intergenic SNP approximately 13 kb from the start of transcription of lecithin retinol acyltransferase (phosphatidylcholine – retinol O-acyltransferase, *LRAT*). We genotyped additional SNPs in this region to test if our significant association extended into the *LRAT *gene. rs727153 is within an LD block flanked by SNPs rs11935519 and rs149225 (Chr4:156,005,695..156,040,821 – HapMap data Rel 21a/phaseII Jan07), which includes *LRAT *(Figure 5). Using a pair-wise approach in Haploview we identified 7 tagging SNPs required to cover the LD block, capturing all common SNPs with an *r*^2 ^> 0.8 and individually genotyping them in our LOAD case – control sample. The results are presented in Table 2. Five SNPs are significantly associated with LOAD in this region, with a SNP in intron 2 of *LRAT*, rs201825 showing the strongest evidence (p-value = 1.7 × 10^-6^). After the addition of extra controls data from the 1958 birth cohort for the 4 SNPs in *LRAT *which have been genotyped in that sample, 3 became more significant (rs201825, p-value = 6.1 × 10^-7^) and the fourth remained unchanged.

**Table 2:** Association analysis of tag SNPs used to cover the LD block containing *LRAT*.

**SNP ID**	**Position (Mb on Chr 4)**	**Minor Allele**	**Minor Allele Freq Cases**	**Minor Allele Freq Controls**	**Minor Allele Freq 1958**	**p Cases vs MRC Cons**	**OR Cases vs MRC Cons (95% CI)**	**p Cases vs Comb Cons**	**OR Cases vs Comb Cons (95% CI)**
Rs12501328	156,019,936	G	0.10	0.08	0.07	0.02661	1.27 (1–1.6)	0.0032	1.32 (1.1 – 1.6)
Rs201825	156,024,540	C	0.52	0.44	0.46	1.65E-06	1.34 (1.2–1.5)	6.12E-07	1.3 (1.2 – 1.4)
Rs201824	156,026,315	T	0.40	0.35		0.00066	1.24 (1.1–1.4)	N/A	N/A
Rs201823	156,026,490	G	0.40	0.35	0.37	0.00042	1.24 (1.1–1.4)	0.0004	1.21 (1.1 – 1.3)
Rs156500	156,034,920	C	0.13	0.11		0.09457	1.17 (1–1.4)	N/A	N/A
Rs156501	156,035,950	A	0.22	0.19	0.19	0.01635	1.2 (1–1.4)	0.0058	1.2 (1.1 – 1.4)
Rs149225	156,040,821	C	0.42	0.40		0.34900	1.06 (0.9–1.2)	N/A	N/A

For descriptions see Table 1. OR – Odds Ratio, N/A – test not conducted.

Analysis of the tagSNPs in this LD block using all possible 2- and 3-marker haplotypes resulted in no evidence for association greater than that reached by rs201825 (data not shown).

Table 3 provides the distribution of the individual genotyping p-values for 109 SNPs outside the *APOE *locus, in addition to the p-value distributions for our three methods used to choose the SNPs for individual genotyping. After individual genotyping, we obtained uncorrected p-values ≤ 0.05 for 74 of the 109 SNPs compared with expected 5.5 for a random selection of SNPs, an enrichment of ~13 fold across all selection methods. The cluster method appeared to generate the highest percentage of significantly associated SNPs (84.8%), at greater levels of significance, identifying the 3 most significant SNPs after individual genotyping. One of the three most significant SNPs (rs3754675) was also in the top 115 SNPs ranked by the "combined" Z-test. The most significant SNP by individual genotyping chosen by the allele frequency method (rs12510838) had a p-value of 0.00024, an order of magnitude less than our most significant SNP chosen by the other methods (Table 1). Thus it appears that the cluster method provided the best way of identifying true associations and that the combined Z-test improves on the identification of single significant SNPs over a simple method of following-up only the highest differences in allele frequencies.

**Table 3:** Distribution of p-values of individually genotyped SNPs (excluding those in the region of *APOE*).

		**P-Value Range**				
**Selection Category**	**N**	**< 0.05**	**0.05 – 0.005**	**5 × 10**^-3^**- 5 × 10**^-4^	**5 × 10**^-4^**- 5 × 10**^-5^	**5 × 10**^-5^**- 5 × 10**^-6^
**ALL SNPs**	109	74 (67.9%)	38 (34.9%)	22 (20.2%)	11 (10.1%)	3 (2.8%)
**CLUSTERS**	33/58	28 (84.8%)	12 (36.4%)	8 (24.2%)	5 (15.2%)	3 (9.1%)
**COMB**	66/115	44 (66.6%)	22 (33.3%)	13 (19.7%)	8 (12.1%)	1 (1.5%)
**FREQ**	46/136	24 (52.2%)	13 (28.3%)	9 (19.6%)	2 (4.3%)	0 (0%)

Selection category gives the method used to choose SNP for individual genotyping (see text for description). N: number of SNPs tested in each selection category. Note: of the 66 SNPs in the COMB category, 11 and 25 SNPs respectively had been identified by the CLUSTER and FREQ methods.

## Discussion

In this study we used DNA pooling to offset the high costs of conducting a GWA study for LOAD. Using Illumina HumanHap300 and Illumina Sentrix HumanHap240S arrays we estimated the allele frequencies of 561,494 SNPs across the genome in pools constructed from 1,082 LOAD cases and 1,239 age-matched controls. We unequivocally identified the *APOE *locus as the major genetic risk factor for the disease. As noted by others this can be seen as a positive control that the pooling method is a viable alternative to individual genotyping in GWA studies for complex disorders [13]. The association of *APOE *with LOAD is well replicated [14,15] and has been observed in a GWA study that used an alternative genotyping platform [16]. Neither the Illumina platform used here nor the Affymetrix system used in that study directly test the SNPs that define the *APOE*-ε4 genotype, highlighting the strength of high density GWA studies to detect indirect association. Our findings are in agreement with others that *APOE *is the major pathogenic locus for LOAD, and that further loci of smaller effect remain to be identified [16,17].

Following pooled genotyping, it is necessary to follow up positive results with individual genotyping to confirm the observed associations. We used three methods to identify SNPs to follow up. The cluster method appeared to generate more true associations than the other methods we used. This is perhaps not surprising, as this method should minimise false positives due to technical artefacts caused by pooled genotyping, as each SNP showing evidence of association was required to be supported by one or more highly significant neighbouring SNP. In contrast, a singleton highly-ranked SNP found by any other method could be due to a technical artefact, no matter how carefully it is filtered. However, the cluster method can miss true positive signals from functional variants that are not in high LD with other tested SNPs. Therefore, we believe that unsupported SNPs have to be followed up as well, unless they are in high LD with other SNPs on the array, which do not support the association. As has been proposed by others, the data presented here suggests that the best way of choosing unsupported SNPs would be to use a statistical test that takes into account technical variation, such as the combined Z-test [11]. We acknowledge that this study has not exhaustively followed up all positive signals – to do this would negate the cost efficiency of the pooling method but it is possible that more significantly associated SNPs could be confirmed by individual genotyping. In fact, we only genotyped individually half of the SNPs that we considered worth following up. However it appears clear that a signal with the strength of association, in terms of a number of highly significant SNPs in a narrow region, as observed at the *APOE *locus, has not been overlooked.

In order to confirm our individual genotyping results, data from the 1958 Birth Cohort were used to form an additional set of controls genotyped with the Illumina platform. These controls have previously been used by the Wellcome Trust Case Consortium for a GWA study for 7 common diseases [18]. There are a number of explanations for our observation that for the majority of SNPs significant in this study the strength of association decreased with additional control data. Firstly, and we believe most likely, the initial findings may have been false positives due to an inflation of the effect size in the original analysis due to sampling variance in the control allele frequencies (i.e. some of the most significant SNPs have the highest sampling variance in either cases or controls). Secondly, the use of unscreened controls in an association study of a disorder of old age is expected to reduce power [19], this is particularly clear in the *APOE *locus. Some degree of genotyping discrepancies between the two platforms used is also possible (Sequenom in our lab and Illumina in the 1958 Birth Cohort), however very unlikely to account for such large differences.

We identified multiple SNP associations (best p-value = 6.12 × 10^-7^) in the gene encoding lecithin retinol acyltransferase (*LRAT*, 4q32.1, **MIM: **604863). This is a highly plausible functional candidate gene for LOAD. *LRAT *plays a prominent role in the Vitamin A (retinoid) cascade by producing retinyl esters, storage forms of retinoid. The retinoid system has been previously implicated in LOAD. Retinoid levels in plasma, serum and brain are lowered in LOAD patients, and the restriction of dietary retinoid in mice results in memory impairment [20,21]. Furthermore disruption of the retinoid signalling pathway in adult rats by a dietary deficiency of vitamin A leads to deposition of amyloid beta in the cerebral blood vessels. There is a down regulation of retinoic acid receptor alpha in the forebrain neurons of the retinoid-deficient rats and a loss of choline acetyl transferase expression, which precedes amyloid beta deposition. In neocortex of pathology samples of patients with Alzheimer's disease, the same retinoic acid receptor alpha deficit in the surviving neurons is observed suggesting that retinoids are important for the maintenance of the adult nervous system and their loss may in part play a role in Alzheimer's disease [22]. Nevertheless, despite the functional plausibility, our genetic findings in *LRAT *fall short of the degree of statistical significance required to provide unequivocal evidence for association, given the large number of comparisons made in a GWA study. Thus our findings, whilst highly suggestive, will require confirmation in independent samples.

## Conclusion

In summary, we have validated the pooling method for GWA studies by both identifying the *APOE *locus, a known risk gene for LOAD, and by observing a strong enrichment for significantly associated SNPs. We have also compared methods for prioritising SNPs for individual genotyping. Finally, we provide evidence for *LRAT *as a novel candidate gene for LOAD. GWA studies with pooled DNA provide a viable, quick and inexpensive approach to identifying susceptibility genes. Inaccuracies, however, mean that some loci that might be detected by individual genotyping will remain undetected.

## Methods

### Subjects

Written informed consent was obtained from all subjects for publication of this case report. A copy of the written consent is available for review by the Editor-in-Chief of this journal. The sample consisted of individuals ascertained from both community and hospital settings in the UK collected as part of the MRC genetic resource for LOAD. Clinical data and DNA samples were collected from 1,082 individuals (71% females) with late-onset AD (LOAD) and 1,239 control subjects (referred to in the text as "MRC controls", 62% females). Age at onset ranged from 60 to 95 years (mean = 75.84 years, SD = 6.79). Controls were matched for age (mean = 76.53 years, SD = 6.33), gender and ethnicity. AD cases and controls described here were ascertained by three collaborating centres: Department of Psychological Medicine, Cardiff University, Cardiff (coordinating centre); Institute of Psychiatry, London; and Cambridge University, Cambridge, as previously described [23]. Ethical approval was obtained from the Multi-centre Research Ethics Committee (MREC), relevant local ethics committees and NHS trusts, in the regions where subjects were recruited.

All cases were Caucasian, of UK origin (parents born in the UK) and diagnosed with probable AD in accordance with the National Institute of Neurological and Communication Disorders and Stroke and the Alzheimer's disease and Related Disorders Associations (NINCDS-ADRDA) clinical diagnostic criteria for AD [24]. All diagnoses were made based on a semi-structured interview with known validity for AD pathology (i.e. positive predictive value of 92–95% [25,26] which included: The Mini Mental State Examination (MMSE) [27]; The Cambridge Mental Disorders of the Elderly Examination (CAMDEX; informant interview) [28]; The Blessed Dementia Scale [29]; The Bristol Activities of Daily Living Scale [30]; Webster Rating Scale [31]; Global Deterioration Scale (GDS) [32]; Cornell Scale for Depression in Dementia [33]; Neuropsychiatric Inventory (NPI) (12-item version) [34]. Interviews were primarily conducted with the AD sufferer's next of kin. Ethical permission was obtained from the Multi-centre Research Ethics Committee, relevant local ethics committees, and National Health Service trusts.

Control subjects were either spouses of AD patients or selected from primary-care practices situated in the same geographical areas as AD patients. All controls were 60 years or above and of UK origin. Control individuals were screened for cognitive decline using the MMSE, and a cut-off score of ≥ 28 was adopted. Assessment of controls also included a section of the Cambridge Mental Disorders of the Elderly Examination and the Geriatric Depression Scale [35]. Exclusion criteria were the presence of dementia, depression, delirium or other illnesses likely to significantly reduce cognitive function.

### DNA Pool Construction

DNA was obtained from blood samples by phenol/choloroform extraction, followed by precipitation in ethanol and storage in TE buffer. DNA quality was assessed by PCR amplification of microsatellite markers under standard conditions, with those samples showing robust amplification being included in pools (1,082 LOAD cases and 1,239 controls). Initial DNA concentrations were determined by UV spectrophotometry using absorbance at 260 nm readings. Dilutions were made using water to bring the concentration of each sample to a target of 20 ng/ul. The concentration of each sample was then determined using the PicoGreen dsDNA Quantitation Reagent (Molecular Probes, Eugene, Ore.) in a Labsystems Ascent Fluoroskan (LifeSciences Int., Basingstoke, UK). Each sample was then diluted to 4 ng/ul (± 0.5 ng/ul), allowed to equilibrate at 4°C for 48 h before another quantification using the PicoGreen method. Samples out of the 4 ng/ul (± 0.5 ng/ul) range were diluted/concentrated, incubated and re-quantified until they were within the required range. Equal volumes of each sample were then added to either a case or control pool using a Biomek^® ^FX Laboratory Automation Workstation (Beckman Coulter, Inc., Fullerton, CA). We chose to make single pools of cases and controls and hybridise them multiple times, rather than construct many small pools and hybridise them on single arrays, as this method has been shown to be more powerful [36].

### Pool validation with the SNaPshot method

In order to test the accuracy of our pool construction, we genotyped 3 significantly associated SNPs in the pools for which we had individual genotype data on the samples used to create the pools (rs11084424, rs157580 and rs157581). SNaPshot genotyping was carried out as previously described [12]. Briefly, forward and reverse primers were designed using primer 3 software . Extension primers for the SNaPshot assay were designed using FP PRIMER 1.0.1 b software . PCR was performed under standard conditions, using 15 ng pooled genomic DNA and HotStar *Taq *DNA polymerase (Qiagen). Primer extension products were run on a 3100 DNA sequencer (Applied Biosystems) and the data were processed by the GeneScan Analysis 3.7 (Applied Biosystems). SNP allele frequencies in DNA pools were estimated from peak heights obtained by using Genotyper 2.5 (PE Biosystems, Cheshire, UK). Estimated allele frequencies from pools were corrected for unequal representation of alleles using the mean of the ratios obtained from four analyses of a heterozygote [37].

### Pooled DNA Genotyping using Illumina Platform

Genome-wide genotyping was performed using Illumina HumanHap300 and Illumina Sentrix HumanHap240S arrays (Illumina Inc., San Diego, CA, USA) according to the manufacturer's protocols. Chips were scanned in standard mode on a BeadStation 500 GX (Illumina) at the University of Bonn and raw data were extracted for statistical analysis with BeadStudio v3.1 software. Because of the expected inter-experiment variation, replicate arrays were genotyped for each pool: four arrays on the Illumina HumanHap300, and 8 arrays on the Illumina Sentrix HumanHap240S array. The reason for using more HumanHap240S arrays is that we first performed the work on HumanHap300, and noticed the need for more replications. Replicate arrays were excluded if the estimated allele frequencies produced a Pearson correlation of r ≤ 0.991 with two or more other replicate arrays of the same pool (i.e. only arrays which correlated at r ≥ 0.992 with each other were retained for analysis). This cut-off was adopted during other pooling work in our department, but in this experiment resulted in the exclusion of data from only a single array (from a control pool) genotyped on the HumanHap300 array.

### Analysis of pooled DNA

Approximation of allele *A *frequencies for each replicate was produced on the basis of the raw data as follows: f_alleleA = Xraw/(Xraw+Yraw), averaged over the number of replicates in each pool (Xraw and Yraw are the intensities of the two dyes Cy5 and Cy3, used to genotype SNPs on the Illumina platform).

### Selection of SNPs for individual genotyping

SNPs from the pooling data were prioritised for individual genotyping based on three different methods, outlined below, since, at the time of this experiment we did not know which method would perform best. For all methods, we excluded rare SNPs (true allele frequency less than 5% in the CEU population of the HapMap, ) and the 5% of SNPs showing the highest technical variability as indicated by the size of the standard deviation amongst measures from the replicate arrays.

#### 1) Cluster Method

We plotted the differences in allele frequencies between cases and controls for each SNP against their position in the genome. We identified 58 clusters where at least two SNPs within 100 kb had a predicted allele frequency difference greater than 5%. One SNP from each cluster was selected for individual genotyping.

#### 2) Allele Frequency Difference Method

Separately for the Illumina HumanHap300 and Illumina Sentrix HumanHap240S arrays (to account for any differences caused by technical artefacts between the two arrays) the pooled data were sorted by predicted allele frequency differences in cases and controls. The top 136 SNPs showing the greatest allele frequency differences (8% difference between cases and controls) and satisfying the above filtering criteria were put forward for potential individual genotyping. SNPs that had previously been flagged as being in a cluster were not included.

#### 3) "Combined Z-test"

The third method was based on p-values estimated using the following statistic which combines experimental and sampling errors, a general description of which has previously been presented [11,36,38]:

$$T_{comb}=\frac{{\left({\bar{f}}^{\left(1\right)}-{\bar{f}}^{\left(2\right)}\right)}^{2}}{v_{1}+v_{2}+\varepsilon_{1}^{2}+\varepsilon_{2}^{2}}.$$

This statistic combines:

a) chi-square statistic *T *for testing differences between two proportions (allele frequencies) in cases and in controls accounting for the sampling variance:

$$T_{1}=\frac{{\left({\bar{f}}^{\left(1\right)}-{\bar{f}}^{\left(2\right)}\right)}^{2}}{v_{1}+v_{2}},$$

where ${\bar{f}}_{k}=\frac{1}{n_{k}}\sum_{i=1}^{n_{k}}f_{i}^{\left(k\right)}$ is the mean of the allele frequencies over *n*_*k *_pool replicates, $v_{k}=\frac{{\bar{f}}_{k}(1-{\bar{f}}_{k})}{2N_{k}}$ is the binomial sampling variance and *N*_*k *_is number of controls and cases respectively (*k *= 1,2).

b) *Z-*statistics for testing the difference in mean allele frequencies between cases and controls:

$$Z=\frac{\left({\bar{f}}_{1}-{\bar{f}}_{2}\right)}{\sqrt{\varepsilon_{1}^{2}+\varepsilon_{2}^{2}}},$$

where $\varepsilon_{k}^{2}=\frac{1}{n_{k}(n_{k}-1)}\sum_{i=1}^{n_{k}}(f_{i}^{\left(k\right)}-{\bar{f}}_{k}{)}^{2}$ is the square of the standard error due to experimental error.

Thus we have taken into account the two single available sources of error: sampling error and experimental error in a simple way which is equivalent to a simplified version of the complex regression model suggested by MacGregor[36].

Excluding 36 SNPs already selected by the allele frequency (25 SNPs) or cluster (11 SNPs) methods, and any filtered ones, 79 of the highest-ranking SNPs identified by the combined Z-test method were put forward for individual genotyping.

### Individual Genotyping

The 273 SNPs chosen by the three methods, (in addition to 5 SNPs at the *APOE *locus which had been identified by all three methods) were presented to the Sequenom Assay Design 3.1 software (Sequenom, San Diego, CA), which selected 130 for genotyping. We deliberately presented about twice as many SNPs to the software, as we wanted to type, in order to maximise the chance of producing well-performing panels.

Genotyping was performed with the MassARRAY and iPlex systems (Sequenom, San Diego, CA) according to the manufacturer's recommendations. Assays were optimized in 30 reference CEU parent – offspring trios, which have been used extensively in the HapMap project. All sample plates contained cases, controls, blanks, CEU and duplicate samples. Quality control (QC) measures included independent double genotyping blind to sample identity and blind to the other rater, and comparison of our CEU genotypes to those in the HapMap database . SNPs showing deviation from Hardy-Weinberg equilibrium (p-value < 0.001 in controls) or with genotyping success rates of less than 90%, or showing differences in genotypes in the CEU samples from those in the HapMap database were excluded from analysis. Individual DNA samples for whom the genotyping success rate across all SNPs was < 75% were also excluded (40 cases and 49 controls).

Of the 130 SNPs chosen from the pooling GWA study, 114 passed QC. Of these 5 were located within 32 kb of *APOE*, 33 were selected from the cluster method, 30 were identified solely by the combined Z-test method and 46 were chosen based on the allele frequency difference.

Calculation of allele frequencies, genotype counts, tests for departure from HWE, allelic association and odds ratio were carried out using PLINK (vers 0.99s, , [39].

Linkage Disequilibrium analysis was conducted using Haploview version 4.0 , [40].

Additional control genotype data were obtained from (, accessed September 2007) which contains genotyping data on the British 1958 Birth Cohort DNA Collection, deposited by the Wellcome Trust Sanger Institute.

## Abbreviations

AD: Alzheimer's Disease; APOE: Apolipoprotein E; DNA: Deoxyribonucleic Acid; GWA: Genome-Wide Association; Kb: kilobase pairs; LD: Linkage Disequilibrium; LOAD: Late-Onset Alzheimer's Disease; LRAT: lecithin retinol acyltransferase; MRC: Medical Research Council; SNP: Single Nucleotide Polymorphism.

## Competing interests

The authors declare that they have no competing interests.

## Authors' contributions

All authors read and approved the final manuscript. GK conceived of and designed the study, analysed pooled genotyping and contributed to writing of manuscript. LG, RS and AM assisted with DNA pool construction and individual genotyping. KD and PH were responsible for patient/control diagnosis and sample collection. SC and AH conducted pooled genome-wide genotyping. VM analysed pooled genotype data, designing combined Z-test and conducted test for association using 1958 control group. MO'D contributed to experimental design and writing of manuscript. JW and MO supervised and contributed to sample collection, interpretation of data and writing of manuscript. RA took the lead in DNA pool construction, writing the manuscript, conducted and analysed individual genotyping.

## Pre-publication history

The pre-publication history for this paper can be accessed here:


